# Association between *C4, C4A*, and *C4B* copy number variations and susceptibility to autoimmune diseases: a meta-analysis

**DOI:** 10.1038/srep42628

**Published:** 2017-02-16

**Authors:** Na Li, Jun Zhang, Dan Liao, Lu Yang, Yingxiong Wang, Shengping Hou

**Affiliations:** 1 Basic Medical College, Chongqing Medical University, Chongqing, China; 2 The First Affiliated Hospital of Chongqing Medical University, Chongqing, China; 3 Chongqing Eye Institute and Chongqing Key Laboratory of Ophthalmology, Chongqing, China

## Abstract

Although several studies have investigated the association between *C4, C4A*, and *C4B* gene copy number variations (CNVs) and susceptibility to autoimmune diseases, the results remain inconsistency for those diseases. Thus, in this study, a comprehensive meta-analysis was conducted to assess the role of *C4, C4A*, and *C4B* CNVs in autoimmune diseases in different ethnic groups. A total of 16 case-control studies described in 12 articles (8663 cases and 11099 controls) were included in this study. The pooled analyses showed that a low *C4* gene copy number (GCN) (<4) was treated as a significant risk factor (odds ratio [OR] = 1.46, 95% confidence interval [CI] = 1.19–1.78) for autoimmune diseases compared with a higher GCN (>4). The pooled statistical results revealed that low *C4* (<4) and low *C4A* (<2) GCNs could be risk factors for systemic lupus erythematosus (SLE) in Caucasian populations. Additionally, the correlation between *C4B* CNVs and all type of autoimmune diseases could not be confirmed by the current meta-analysis (OR = 1.07, 95% CI = 0.93–1.24). These data suggest that deficiency or absence of *C4* and *C4A* CNVs may cause susceptibility to SLE.

The complement system, which is involved in both innate and adaptive immunity, is characterized by triggered-enzyme cascades activated by alternative, lectin or classical pathways^1^. As a necessary and important component of the complement system, complement component *C4* plays a pivotal role in the activation of immune defenses and the clearance of immune complexes or apoptotic debris *in vitro* and *in vivo*^2^,^3^. If a structural variation such as fragmentary deficiency or deletion of a complement *C4* gene occurs, it could cause an aberrant immune response and an autoimmune or inflammatory disorder^4^. Genetically, the complement component *C4* gene is mapped in the class III region of the major histocompatibility complex (MHC) on chromosome 6p21.3 and there are two isotypes of *C4* gene products, which are acidic *C4A* and basis *C4B*^5^. The *C4A* and *C4B* isotypes can be distinguished by four specific amino acids at positions 1101–1106 (or 1120–1125 if the protein numbering starts at the initiation codon for Methionine), which were the results of five nucleotide polymorphisms^6^,^7^. *C4A* prefers to bind to amino groups of immune complexes, while *C4B* binds to hydroxyl groups when activated^8^,^9^. Cumulative research has confirmed that gene expression as well as *C4, C4A*, and *C4B* CNVs could affect the strength of an individual’s immunity and susceptibility to autoimmune diseases^8^,^10^,^11^,^12^. The link between complement component C4 complete protein deficiency and autoimmune diseases was first observed among patients with systemic lupus erythematosus (SLE) in 1974^13^.

CNVs in the human genome belong to a significant sequence variation, indicating that large stretches of DNA may exist in a number of different forms in different individuals^14^. CNVs in genes performing functions related to immunity can increase immunological diversity and genetic predisposition to autoimmune diseases^15^. It has been reported that partial *C4* deficiency is one of the most frequent immunoprotein deficiencies in Homo sapiens^10^. Additionally, both *C4A* and *C4B* have various GCNs according to the Database of Genomic Variants (http://projects.tcag.ca/variation). The *C4* gene encodes for either *C4A* or *C4B*, so the GCN of total *C4* is equal to the sum of the GCNs of *C4A* and *C4B*^16^. CNV patterns in individuals belonging to different racial groups vary in terms of the number and size of *C4* genes. *C4* exists as two isoforms encoded by different genes, *C4A* and *C4B.* Together with three neighboring genes, *C4* forms a genetic unit called the RCCX module (*RP-C4A-CYP21-TNX* or *RP-C4B-CYP21-TNX*). In the human diploid genome, the total *C4* GCN varies from 2 to 6, with 4 being the most common. *C4A* GCN varies from 0 to 5, while that of *C4B* varies from 0 to 4, with 2 being the most common for both genes^17^. In Caucasian populations from the US and Europe, ~80% of individuals have three or four *C4* GCNs, 20% have five or six, and <2% have only two^8^,^10^. In Chinese populations, about 60% of individuals have four *C4* GCNs, and more than 63% have two *C4A* and *C4B* GCNs^18^,^19^. The most common GCNs of total *C4, C4A* and *C4B* in all racial groups are four, two, and two, respectively.

Deficiencies of *C4* are strongly associated with increased risk of developing SLE^20^. However the association has failed to be confirmed in certain autoimmune diseases^18^. This inconsistency may be attributed to studies’ small sample size, genotyping method or different pathogeneses for different diseases and so on. Meta-analysis is an effective way to synthesize primary data from independent studies and generate more adequate and accurate statistical conclusions according to pooled analyses and results. We summarized current knowledge related to the role of *C4, C4A*, and *C4B* CNVs in SLE and other systemic autoimmune diseases and determined whether *C4* genes are a genetic master key to autoimmunity. Subgroup analyses focusing on disease type and ethnicity were conducted to investigate potential sources of heterogeneity in the included studies.

## Results

### General characteristics of studies

**Figure 1:**
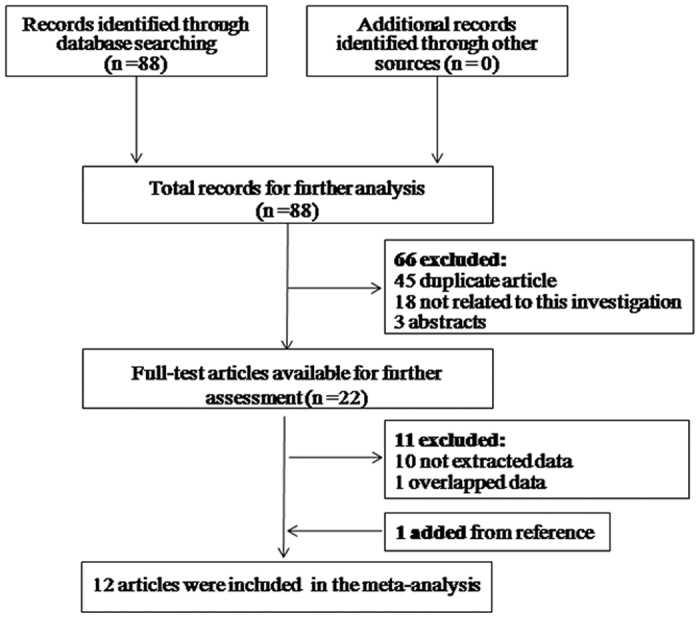
Flow diagram presenting the result of literature searching process in meta-analysis.

The literature selection process is shown in Fig. 1. A total of 88 articles were initially identified using PubMed, Embase, Web of Science, and the China National Knowledge Infrastructure (CNKI). Sixty-six articles were excluded because they were duplicate studies, abstracts or not related to CNVs in autoimmune diseases. For detailed evaluation, another 10 articles without sufficient data and 1 article with overlapping data were ruled out. Additionally, a case-control study related to this subject was found by carefully screening the reference lists of each included article. Finally, 16 case-control studies from 12 articles were included in this meta-analysis. The studies’ characteristics and the extracted data are summarized in Table 1 17,18,19,21,22,23,24,25,26,27,28,29. As shown in Table 1, 8 studies focused on SLE, and 8 were mainly involved with GD, Crhon’s disease (CD), rheumatoid arthritis (RA), juvenile dermatomyositis (JDM), and uveitis. To identify differences between racial groups, among of 16 studies, 7 studies studying Asian populations and 9 studying Caucasian populations were enrolled in this analysis. Regarding genotyping method, a TaqMan-based quantitative real-time polymerase chain reaction (TaqMan) was applied in most studies (n = 10).

### Bias assessment of the included studies

The potential bias assessment of the included studies is presented in Table 2. Overall, the quality of the included studies was consistently good. Of the studies, there was no bias regarding selection, controls, genotyping controls, confounding, multiple tests, or selective outcome reports.

### Meta-analysis results

**Figure 2:**
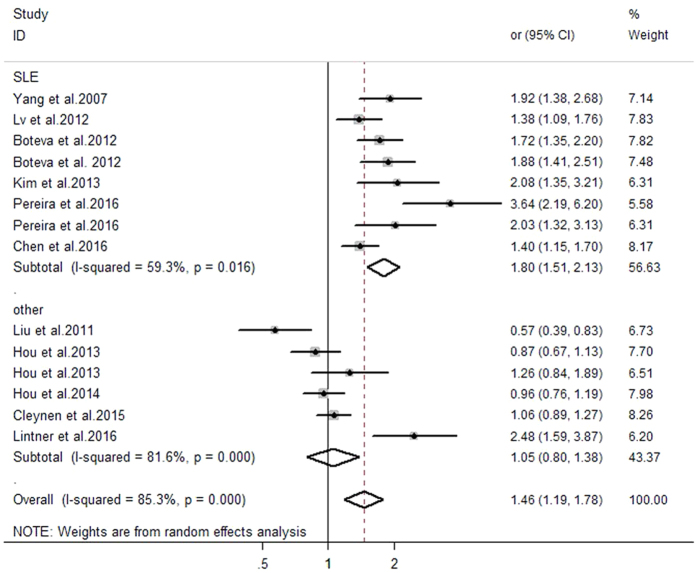
Evaluation of the association between *C4* gene CNVs with autoimmune diseases.

**Figure 3:**
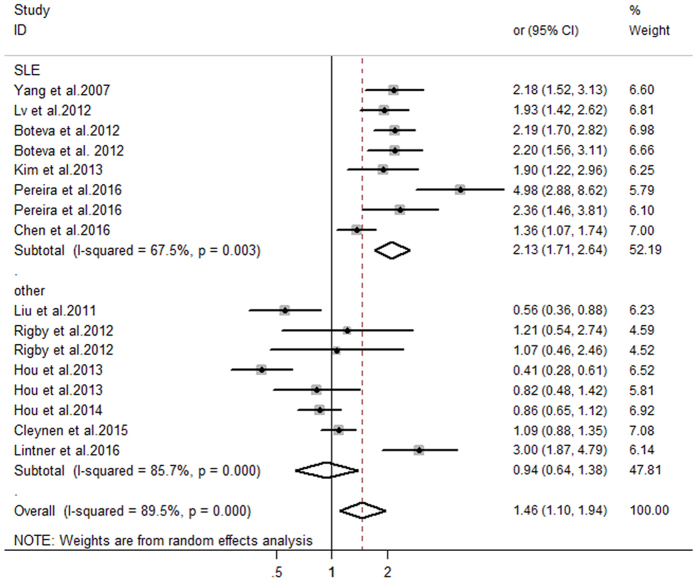
Assessment of the association between *C4A* gene CNVs with autoimmune diseases.

**Figure 4:**
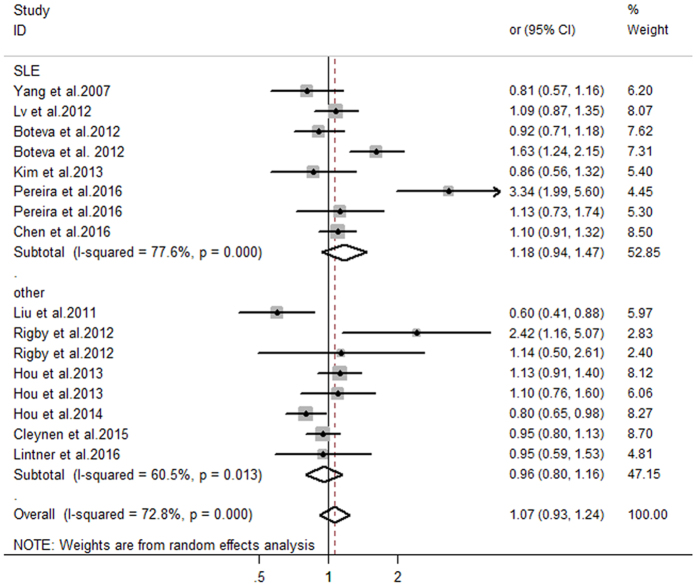
Estimation of the association between *C4B* gene CNVs with autoimmune diseases.

The relationship between *C4, C4A*, and *C4B* CNVs and autoimmune diseases was investigated, and the detailed results of pooled odds ratios (ORs) and a stratification analysis are presented in Table 3. The pooled analyses suggested significant between-study heterogeneity among the three genes (*C4*: I^2^ = 85.3%, P_H_ < 0.001; *C4A*: I^2^ = 89.5%, P_H_ < 0.001; *C4B*: I^2^ = 72.8%, P_H_ < 0.001). Hence, the meta-analysis detected a random-effect model. As presented in Fig. 2, there was a significant association between *C4* GCN and autoimmune diseases. Individuals with low *C4* copy numbers (<4) were more likely to develop an autoimmune disease (pooled OR = 1.46, 95% CI: 1.19–1.78). In the subgroup analysis involving disease type, the risk of SLE was clearly increased in individuals with low *C4* GCNs (pooled OR = 1.80, 95% CI: 1.51–2.13). Moreover, the subgroup analysis involving ethnicity revealed that low *C4* GCNs were significantly associated with the risk of autoimmune diseases among Caucasian individuals (pooled OR = 1.91, 95% CI:1.42–2.56), while such an association was not confirmed in an Asian population (pooled OR = 1.13, 95% CI: 0.88–1.45). Furthermore, the results suggested that low *C4A* GCNs (<2) are more associated with apparent risk for autoimmune diseases compared with higher GCNs (≥2) (pooled OR = 1.46, 95% CI: 1.10–1.94) (Fig. 3). The subgroup analysis involving both disease type and ethnicity indicated that low *C4A* GCNs were significantly associated with susceptibility to SLE and a Caucasian population (pooled OR = 2.13, 95% CI: 1.71–2.64; pooled OR = 2.05, 95% CI: 1.49–2.83, respectively). No significant association between low *C4B* GCNs (<2) and autoimmune diseases was found in this meta-analysis (OR = 1.07, 95% CI: 0.93–1.24) (Fig. 4).

The fixed effect model was applied to these data in accordance with Chen *et al*.’s report^30^. In the subgroup analysis involving disease type, we found a significantly increased risk for SLE among carriers with low *C4* or *C4A* GCNs (pooled OR = 1.68, 95% CI: 1.51–1.86; pooled OR = 1.99, 95% CI: 1.77–2.24, respectively). Finally, the studies were stratified by ethnicity, which found an apparently increased risk of autoimmune diseases associated with low *C4* or *C4A* GCNs in Caucasians (pooled OR = 1.57, 95% CI: 1.41–1.75; pooled OR = 1.83, 95% CI: 1.62–2.06, respectively). These results suggest that, regardless of the random effect model or fixed effect model used for meta-analysis, the pooled data was consistent, believable, and stable.

### Heterogeneity test and sensitivity analysis

As suggested in Table 3, there was significant heterogeneity between studies in terms of GCNs (p < 0.05). The results of our subgroup analysis confirmed that disease type and ethnicity were the main sources of heterogeneity. Additionally, a sensitivity analysis was conducted to evaluate the effect of individual studies on the pooled ORs by sequentially omitting each study. The pooled ORs were not affected by excluding any study (data not shown).

### Publication bias

**Figure 5:**
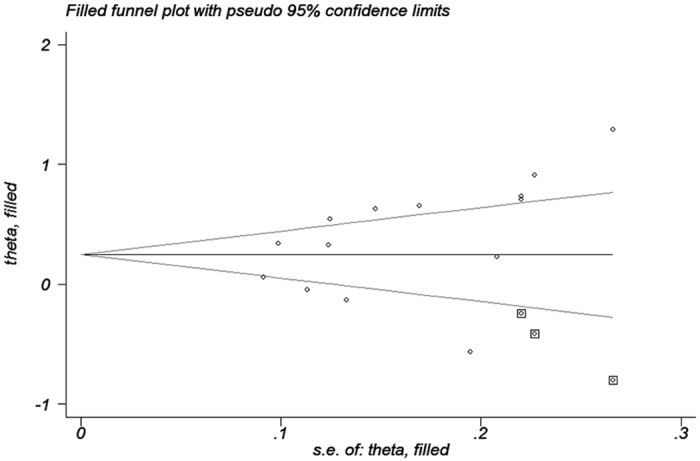
Evaluation of the publication bias between *C4* gene CNVs with autoimmune diseases.

Begg’s funnel plots and Egger’s regression tests were applied to determine the potential publication bias for *C4, C4A*, and *C4B* CNVs. As presented in Table 4, there was no obvious publication bias among these detection. In order to validate whether there was potential publication bias regarding *C4*, we performed a funnel plot using the trim and fill method. LnOR and 95% CI were 0.376 (0.173, 0.579) and 0.246 (0.042, 0.450), respectively, before and after applying the trim and fill method, which indicated that publication bias was present in the meta-analysis (Fig. 5).

## Discussion

In our meta-analysis of 8663 cases and 11099 controls in 16 studies from 12 articles, we drew a general conclusion that *C4* and *C4A* CNVs are tightly associated with autoimmune diseases, especially with SLE. Individuals with low GCNs of *C4* (<4) or *C4A* (<2) are predisposed to autoimmune disorders in the presence of environmental triggers. Consistent with previous studies, both *C4* and *C4A* CNVs were regarded as pivotal genetic factors in the pathogenesis of SLE. Furthermore, our meta-analysis demonstrated that low GCNs of *C4* (<4) or *C4A* (<2) could lead to increased risk of autoimmune diseases among Caucasian populations. In other words, differences in the association between different ethnicities may result from other factors, such as geography, socioeconomic development, or race.

Complement *C4* plays an essential role in innate and adaptive immune responses, which are involved in the classical and mannose-binding lectin complement activation pathways and help to direct against external attacks such as microbial infection, clearance of immune complexes, and removal of apoptotic cells^31^,^32^. Human *C4* protein is encoded by two polymorphic genes, *C4A* and *C4B*, which are located in the MHC on chromosome 6. *C4* deficiency and dysfunction are linked to the pathogenesis of many autoimmune and inflammatory diseases^33^. Several studies investigating different ethnic groups have shown that *C4* deficiency may be one of the most penetrant genetic risk factors of SLE^4^,^21^,^23^. The results of the current meta-analysis were consistent with earlier observations.

*C4A* and *C4B* are two isotypes of *C4*. Although they share >99% of their amino acid sequences, their chemical reactions to substrates are remarkably different. *C4A* tends to combine amino group-containing antigens or immune complexes, while *C4B* combines hydroxyl group-containing antigens. Because *C4A* more efficiently handles immune complexes, deficiency of *C4A* while *C4B* affects the development of SLE^34^,^35^.

It is generally accepted that CNVs are a genetic determinant of phenotypic variation^9^. A CNV is a type of structural variation in which large segments of DNA are altered due to duplication, deletion, insertion, inversion, or complex combination or rearrangement^36^. Loci involved in immunity are prone to CNV, leading to differences in the intrinsic strength of the immune system and variations in susceptibility to immune disorders^8^,^37^. Haraksingh *et al*.^38^ found that CNVs may affect gene dosage due to various copies of a certain gene that is present in the genome. Yang *et al*.^8^ has reported a strong, positive correlation between *C4* GCN and serum *C4* protein concentration as well as *C4A* and *C4B* gene dosage and serum *C4A* and *C4B* concentration. In their study, a low GCN group (for *C4,* n < 4; for *C4A*, n < 2) had a significantly lower serum protein concentration than that of a medium GCN group (for *C4*, n = 4; for *C4A*, n = 2) and a high GCN group (for *C4*, n > 4; for *C4A*, n > 2)^8^. Since *C4* plays a pivotal role in innate and adaptive immune responses, individuals with low GCNs of *C4* (<4) or *C4A* (<2) could show reduced serum *C4* or *C4A* concentrations, resulting in dysfunction in the ability to resist microbial infection, clear immune complexes, and remove apoptotic cells. Thus, low *C4* CNV is a risk factor for SLE. As we know, SLE, RA, BD, and AS are immune-mediated autoimmune diseases. In this meta-analysis, we planned to summarize the current knowledge related to the relationship between *C4* CNVs and SLE and other systemic autoimmune diseases and to determine whether *C4* is a genetic master key to autoimmunity. Liu *et al*.^22^ revealed that individuals with less than 4 copies of *C4* and those with less than 2 copies of *C4A* and *C4B* tended to be at less risk for GD. Liu *et al*.^22^ also found that less than 2 copies of *C4A* may be associated with high risk for vitiligo in patients with GD. Hou *et al*.^19^ found that patients with Vogt-Koyanagi-Harada (VKH) syndrome have decreased frequency of high *C4* GCNs (>4) and decreased expression of serum C4 in patients. Hou *et al*.^18^ indicated that high GCNs (>2) of *C4A* lead to risk for BD but not acute anterior uveitis by modulating the expression of *C4A* and enhancing IL-6 production. In another study, Lintner *et al*.^28^ discovered that there were significant differences in the distribution of lower *C4A* and *C4* GCNs (<2 and <4, respectively) among JDM patients and controls, suggesting that complement *C4A* deficiency appeared to be an important risk factor for JDM. Furthermore, Rigby *et al*.^26^ demonstrated that *C4B* deficiency resulted in increased risk for RA and had broad implications for the pathogenesis of RA. However, data from pooled analyses of *C4* and non-SLE autoimmune diseases are relatively insufficient. Additionally, data from several published articles were only done by single method without validation using the other methods and therefore further studies with different methods and larger samples including individuals from different ethnic backgrounds would help reveal the role of *C4* in GD, uveitis, JDM, and RA.

Some limitations of the current meta-analysis of the potential relationship between *C4, C4A*, and *C4B* CNVs and autoimmune diseases need to be addressed. First, heterogeneity among ethnic groups or disease types was discovered when the association between *C4, C4A*, and *C4B* CNVs and autoimmune diseases was investigated. However, based on the results of the sensitivity analysis, it is clear that the overall results were not affected by heterogeneity. Second, an obvious publication bias was detected in the comparison between *C4* CNV and autoimmune diseases. Third, the size of the patient and control groups was relatively small in each study; therefore, future studies should employ a much larger sample size and include individuals from different ethnic populations. Fourth, the effects of common confounding factors, including sex, age, and medical condition, were not assessed in the present study because of insufficient data. Fifth, autoimmune diseases at various sites may vary widely in terms of the etiology of pathology, including genetic factors, immunologic factors, and environmental factors. Finally, the electronic databases from which we selected eligible studies were listed in English and Chinese; thus, the meta-analysis may have a language bias.

In conclusion, the present meta-analysis provides evidence-based pooled data revealing a significant association between low *C4* or *C4A* CNVs and susceptibility to autoimmune diseases, especially for Caucasian individuals with SLE.

## Materials and Methods

### Literature search

The Preferred Reporting Items for Systematic Reviews and Meta-Analyses (PRISMA) criteria were using as a guide for this meta-analysis^39^. A systematic search of PubMed, Embase, Web of Science, the China Biomedical database, and CNKI was conducted with the following keywords: C4 OR complement C4 OR complement component 4 OR C4A OR complement C4A OR complement component 4A OR C4B OR complement C4B OR complement component 4B AND copy number variation OR copy number variations OR CNV OR CNVs OR gene copy number OR GCN AND autoimmune disease OR autoimmune disorder OR autoimmunity. These in silico literature searches were restricted to human studies, and we used studies published until September 20, 2016. No language restrictions were imposed. The reference lists of the retrieved articles were manually searched in order to identify more relevant studies.

### Inclusion and exclusion criteria

Studies selected from electronic databases were included when they met the following criteria: (1) focus on the CNVs of total *C4, C4A*, or *C4B* in relation to autoimmune diseases; (2) a case-control study design; (3) presence of enough data to calculate an OR and corresponding 95% CI; and (4) inclusion of patients diagnosed according to international standards. The following items were regarded as the primary exclusion criteria: (1) focus on families or twins rather than random general populations; (2) inclusion of individuals with overlapping investigated diseases; (3) a case study design; (4) insufficient data applied after emailing the relevant corresponding author. When several articles with the same types of patients were identified, the most detailed article with the largest sample size and most comprehensive analysis was included in the meta-analysis.

### Data extraction

Data from the retrieved studies were extracted independently by two authors (N.L. and J.Z.). The following information was collected from each eligible study: first author, year of publication, country of origin, ethnicity of subjects, type of disease, sample size, genotyping methods, and information about GCN distribution in cases and controls. Two authors carefully screened the collected data and reached agreement in all respects. When the authors disagreed, a third reviewer (D.L.) weighed the arguments and then helped reach a consensus.

### Quality assessment

Evaluation of the quality of extracted studies was also performed by two authors (N.L. and J.Z.) based on the HuGENet Handbook^40^. Six bias assessment items referring to the association between genes and disease were derived from this handbook, including bias in selection of cases, bias in selection of controls, bias in genotyping cases, bias in genotyping controls, bias in population stratification, confounding bias, multiple tests, and selective outcome reports. The quality of every item was labeled as “Yes” or “No,” while “Unclear” was used if there was not enough information to make a determination. A correction and review were performed independently by the investigator (D.L.) if the two coauthors dissented. Consensus regarding all labels was achieved after discussion.

### Statistical analysis

ORs and the corresponding 95% CIs were estimated to evaluate the amount of correlation between *C4, C4A*, and *C4B* CNVs and susceptibility to autoimmune diseases. The diversity of genotypes (for total *C4*, the number of subjects with <4 GCNs compared to those with ≥4 or >4, and for *C4A* and *C4B*, the number of subjects with <2 GCNs compared to those with ≥2 or >2) among cases and healthy controls was compared. The heterogeneity between studies was estimated and measured by Cochran’s Q statistic as well as the I^2^ statistic. When the p value of chi-square statistic was less than 0.05 or the I^2^ value was more than 50%, the random-effect model was adopted for meta-analysis, indicating that heterogeneity existed across studies^41^. In addition, a goodness of fit test proposed by Chen *et al*.^30^ was used to explore the adequacy of the model for our systematic meta-analysis. Sensitivity analyses were performed to assess the effect of individual studies on pooled ORs by omitting each study in turn. Publication bias was estimated by inspecting Begg’s funnel plots^42^ and Egger’s regression test^43^. Statistical data was analyzed using STATA 12.0 software (StataCorp LP, College Station, Texas, USA). A significant difference was estimated at p < 0.05 (a two-tailed p value). The final conclusions of the study were independently validated by two authors (J. Z. and S. H.).

## Additional Information

**How to cite this article:** Li, N. *et al*. Association between *C4, C4A*, and *C4B* copy number variations and susceptibility to autoimmune diseases: a meta-analysis. *Sci. Rep.* **7**, 42628; doi: 10.1038/srep42628 (2017).

**Publisher's note:** Springer Nature remains neutral with regard to jurisdictional claims in published maps and institutional affiliations.

## Figures and Tables

**Table 1:** The General Characteristics of All Studies Included in this Meta-Analysis.

Refs	Year	Country	Ethnicity	Case/Control	Disease	Typing teaching	Study design
Liu *et al*.^22^	2011	China	Asian	624/160	GD	TaqMan	Case-control,sex-, ethnic-, matched
Lv *et al*.^23^	2012	China	Asian	924/1007	SLE	TaqMan	Case-control,age-, ethnic-, matched
Kim *et al*.^21^	2013	Korea	Asian	308/307	SLE	TaqMan	Case-control,age-, ethnic-, matched
Yang *et al*.^17^	2007	America	Caucasian	216/517	SLE	RFLP	Case-control,age-, ethnic-, matched
Boteva *et al*.^24^	2012	UK	Caucasian	501/719	SLE	Four-digit genotyping	Case-control, ethnic-, matched
Boteva *et al*.^24^	2012	Spanish	Caucasian	464/449	SLE	Four-digit genotyping	Case-control, ethnic-, matched
Cleynen *et al*.^25^	2015	Belgium	Caucasian	1887/1032	CD	TaqMan	Case-control, ethnic-, matched
Hou *et al*.^19^	2014	China	Asian	1027/2083	VKH	TaqMan	Case-control,age-, ethnic-, matched
Rigby *et al*.^26^	2012	America	Caucasian	160/51	RA	Southern blot analyses	Case-control,age-, ethnic-, matched
Rigby *et al*.^26^	2012	America	Caucasian	88/51	non-RA rheumatism	Southern blot analyses	Case-control,age-, ethnic-, matched
Pereira *et al*.^27^	2016	Brazil	Caucasian	90/200	jSLE	TaqMan	Case-control, ethnic-, matched
Pereira *et al*.^27^	2016	Brazil	Caucasian	170/200	SLE	TaqMan	Case-control, ethnic-, matched
Hou *et al*.^18^	2013	China	Asian	905/1238	BD	TaqMan	Case-control,age-, ethnic-, matched
Hou *et al*.^18^	2013	China	Asian	205/1238	AS + AAU+	TaqMan	Case-control,age-, ethnic-, matched
Lintner *et al*.^28^	2016	America	Caucasian	95/500	JDM	Southern blot analyses	Case-control,age, ethnic-, matched
Chen *et al*.^29^	2016	China	Asian	999/1347	SLE	TaqMan	Case-control, ethnic-, matched

**Table 2:** Assessment of potential bias in enrolled studies.

Year	First author	Bias in selection of cases	Bias in selection of controls	Bias in genotyping controls	Bias in population stratification	Confounding bias	Multiple test and Selective outcome reports
2011	Liu *et al*.	NO	NO	NO	NO	NO	NO
2012	Lv *et al*.	NO	NO	NO	NO	NO	NO
2013	Kim *et al*.	NO	NO	NO	NO	NO	NO
2007	Yang *et al*.	NO	NO	NO	NO	NO	NO
2012	Boteva *et al*.	NO	NO	NO	NO	NO	NO
2015	Cleynen *et al*.	NO	NO	NO	NO	NO	NO
2014	Hou *et al*.	NO	NO	NO	NO	NO	NO
2012	Rigby *et al*.	NO	NO	NO	NO	NO	NO
2016	Pereira *et al*.	NO	NO	NO	NO	NO	NO
2013	Hou *et al*.	NO	NO	NO	NO	NO	NO
2016	Lintner *et al*.	NO	NO	NO	NO	NO	NO
2016	Chen *et al*.	NO	NO	NO	NO	NO	NO

**Table 3:** Main Results of Pooled ORs and Analysis of *C4*,*C4A* and *C4B* CNV with Autoimmune Diseases in this Meta-Analysis.

		<4 VS ≥ 4 *C4*			<2 VS ≥ 2 *C4A*			<2 VS ≥ 2 *C4B*		
Classification	N(Case/Control)	OR	95%CI	P_H_	OR	95%CI	P_H_	OR	95%CI	P_H_
All disease		1.46	(1.19, 1.78)	<0.001	1.46	(1.10, 1.94)	<0.001	1.07	(0.93, 1.24)	<0.001
SLE	8(3672/4746)	1.80	(1.51, 2.13)	0.016	2.13	(1.71, 2.64)	0.003	1.18	(0.94, 1.47)	<0.001
Other	8(4991/6353)	1.05	(0.80, 1.38)	<0.001	0.94	(0.64, 1.38)	<0.001	0.96	(0.80, 1.16)	0.013
Ethnicity										
Asian	7(4992/7380)	1.13	(0.88, 1.45)	<0.001	0.98	(0.65, 1.49)	<0.001	0.96	(0.82, 1.12)	0.021
Caucasian	9(3671/3719)	1.91	(1.42, 2.56)	<0.001	2.05	(1.49, 2.83)	<0.001	1.25	(0.96, 1.63)	<0.001

P_H_: P value for heterogeneity.

**Table 4:** Bias between *C4*,*C4A* and *C4B* CNV with Autoimmune Diseases in this Meta-Analysis.

Gene	Number of publication	Publication bias	
		Begg’s test	Egger’s test
<4 VS ≥ 4 *C4*	14	0.063	0.096
<2 VS ≥ 2 *C4A*	16	0.893	0.720
<2 VS ≥ 2 *C4B*	14	0.444	0.300
