# Corrigendum: Association between *C4, C4A*, and *C4B* copy number variations and susceptibility to autoimmune diseases: a meta-analysis

**DOI:** 10.1038/srep46785

**Published:** 2017-05-03

**Authors:** Na Li, Jun Zhang, Dan Liao, Lu Yang, Yingxiong Wang, Shengping Hou

Scientific Reports 7: Article number: 4262810.1038/srep42628; published online: 02 16 2017; updated: 05 03 2017

The Acknowledgements section in this Article is incomplete.

“This work was supported by National Natural Science Foundation Project (81522013), Chongqing Outstanding Youth Grant (cstc2014jcyjjq10005), Chongqing Key Laboratory of Ophthalmology (CSTC, 2008CA5003), Chongqing Science & Technology Platform and Base Construction Program (cstc2014pt-sy10002), National Key Clinical Specialties Construction Program of China and Research and Cultivation Foundation Project of Chongqing Medical University (201407)”.

Should read:

“This work was supported by National Natural Science Foundation Project (81522013, 31571190), Chongqing Outstanding Youth Grant (cstc2014jcyjjq10005), Chongqing Key Laboratory of Ophthalmology (CSTC, 2008CA5003), Chongqing Science & Technology Platform and Base Construction Program (cstc2014pt-sy10002), National Key Clinical Specialties Construction Program of China, Chongqing Graduate Student Research Innovation Project (CYB16096) and Research and Cultivation Foundation Project of Chongqing Medical University (201407)”.

